# Recent Advances in Conjugated Polymers for Light Emitting Devices

**DOI:** 10.3390/ijms12032036

**Published:** 2011-03-21

**Authors:** Mohamad Saleh AlSalhi, Javed Alam, Lawrence Arockiasamy Dass, Mohan Raja

**Affiliations:** 1 King Abdullah Institute for Nanotechnology, P.O. Box 2455, King Saud University, Riyadh 11451, Saudi Arabia; E-Mails: malsalhi@ksu.edu.sa (M.S.A.); ldass@ksu.edu.sa (L.A.D.); rmohan@ksu.edu.sa (M.R.); 2 Physics and Astronomy Department, College of Science, P.O. Box. 2455, King Saud University, Riyadh 11451, Saudi Arabia

**Keywords:** fluorescent polymers, conjugated polymers, organic light emitting diodes, polymer laser devices, semiconductor

## Abstract

A recent advance in the field of light emitting polymers has been the discovery of electroluminescent conjugated polymers, that is, kind of fluorescent polymers that emit light when excited by the flow of an electric current. These new generation fluorescent materials may now challenge the domination by inorganic semiconductor materials of the commercial market in light-emitting devices such as light-emitting diodes (LED) and polymer laser devices. This review provides information on unique properties of conjugated polymers and how they have been optimized to generate these properties. The review is organized in three sections focusing on the major advances in light emitting materials, recent literature survey and understanding the desirable properties as well as modern solid state lighting and displays. Recently, developed conjugated polymers are also functioning as roll-up displays for computers and mobile phones, flexible solar panels for power portable equipment as well as organic light emitting diodes in displays, in which television screens, luminous traffic, information signs, and light-emitting wallpaper in homes are also expected to broaden the use of conjugated polymers as light emitting polymers. The purpose of this review paper is to examine conjugated polymers in light emitting diodes (LEDs) in addition to organic solid state laser. Furthermore, since conjugated polymers have been approved as light-emitting organic materials similar to inorganic semiconductors, it is clear to motivate these organic light-emitting devices (OLEDs) and organic lasers for modern lighting in terms of energy saving ability. In addition, future aspects of conjugated polymers in LEDs were also highlighted in this review.

## 1. Introduction

In 1977, Alan J. Heeger, Alan MacDiarmid and Hideki Shirakawa found out that a thin film of polyacetylene could be oxidized with iodine vapors, turning the material into a conductor. This sensational finding earned them the 2000 Nobel Prize in Chemistry. Thanks to their pioneering discoveries, this versatile plastic conductor, which is a type of polymer with extended conjugated backbone, is now researched in a large international field with significant academic and industrial activities. In the 1980s, the future for conjugated polymers in commercial development further attracted tremendous scientific and industrial interest due to their potential in achieving the goal of light emitting device technology that is economically viable for solid-state lighting and displays, which offer significant gains in power efficiency, color quality, and life time at lower cost and less environmental impact than traditional incandescent and fluorescent lighting [1–9]. The features of conjugated polymers that made them particularly promising to light emitting devices are the large nonlinear optical figure of merit, electronic structure, energy band gap, high optical damage thresholds, ultrafast optical responses and architectural flexibility along with processing advantages and mechanical properties of polymers [10–14]. Basically, conjugated polymers are organic macromolecules which consist of at least one chain of alternating double- and single-bonds. They derive their semiconducting properties from having the extensive delocalization of π-electron bonding along the polymer chain and this delocalized π electron system makes them capable of absorbing sunlight, creating photogenerated charge carriers and transporting these charge carriers. Moreover, these significant properties can be altered by the inclusion of functional side groups as well as substitution of the intractable conducting polymers backbone with alkyl and alkoxy substituents [15–21].

### 1.1. At a Glance: Scientific Interest in Conjugated Polymers

**Figure 1.**
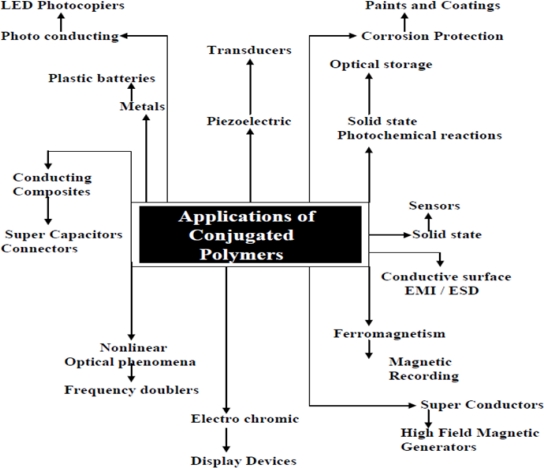
Conjugated polymer applications.

The recent and rapid development of materials and devices designed for efficient manufacture have endowed the concept of “polymer light” with the optical, electrical, and mechanical characteristics that truly make it a disruptive technology within the display and lighting industries, in that it is compatible with conventional device replacement and offers new opportunities for exploitation [22–27]. Concerning “polymer light”, the discovery in Cambridge of electroluminescence (EL), which is the emission of light when excited by flow of an electric current, in conjugated polymers has provided a new impetus to the development of light-emitting devices (LEDs) for display and has a promising future for other purpose [28]. The major scientific interests in conjugated polymers for material scope and development are shown in Figure 1:

1950s—steady work on crystalline organic states.
1970s—organic photoconductors (Xerography).
1980s—organic non-linear optical materials.
1987—Kodak first published the efficient organic light-emitting devices (OLED). 1988—Polymer field-effect transistor demonstrated.
1990—Cambridge groups publish the first polymer light-emitting diodes (PLED). 1995—Efficient polymer photovoltaic diodes demonstrated.
2000—World’s first full color ink-jet printed PLED display.
2009—Google, Nokia, Samsung selling millions of phones with touch OLED screen, first OLED lighting panel.
2010—Osram Opto Semiconductors has introduced Orbeos, its first OLED light source.

## 2. Recent Literature Survey

**Figure 2.**
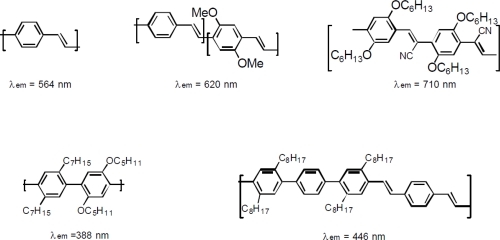
Conjugated polymeric light emitting materials.

The first use of conjugated polymers was as conductors in applications varying from battery electrodes to long-term stable polymer capacitors [29–37]. However, in the late 1980s, a group headed by Prof. Richard Friend of Cambridge University, UK, discovered a new application for these polymers, namely as an electroluminescent device. His work showed that the semiconductive conjugated polymer poly (p-phenylenevinylene) (PPV) showed electroluminescent characteristics if an appropriate choice of contact layers was made. Since then, tremendous progress in this field has been made in many aspects such as fundamental science in order to realize commercial applications, opportunities for processing, device structures and performances in addition to new conjugated polymers and their derivatives as electroluminescent materials [38–51] where some of promising electroluminescent conjugated polymers and their derivatives are shown in Figure 2. Many excellent research papers, patents and reviews were published concerning these aspects, which promoted conjugated polymers to be promising EL materials [52–57].

Poly (*p*-phenylene vinylene) (PPV, or polyphenylene vinylene) is a bright yellow, fluorescent conjugated polymer. Its emission maxima at 551 nm (2.25 eV) and 520 nm (2.4 eV) are in the yellow-green region of the visible spectrum. PPV is the only polymer of this type that has so far been successfully processed into a highly ordered crystalline thin film. PPV and its derivatives are conducting polymers of the rigid-rod polymer family. They are the only conjugated polymers that have been successfully processed in film with high levels of crystallinity. PPV is easily synthesized in good purity and high molecular weight. Although insoluble in water, its precursors can be manipulated in aqueous solution. The small optical band gap and its bright yellow fluorescence make PPV a candidate in many electronic applications such as light-emitting diodes (LED) and photovoltaic devices. Moreover, PPV can be easily doped to form electrically conductive materials. Its physical and electronic properties can be altered by the inclusion of functional side groups. Although PPV is a very promising light emitting conjugated polymer, some processing problems exist. The unsubstituted form of PPV is insoluble in organic solvents, with proper chemical modifications of the polymer backbone it can be dissolved in organic solvents and their physical and electronic properties can also be altered by the inclusion of functional side groups [58–63].

The literature survey revealed that very insightful reviews of the general character of conjugated polymers have been presented by Holmes *et al.* in 1998 [64], Friend *et al.* in 1992 [65], and Bäuerle *et al.* in 2002 [66]. Among the recent papers, one of the most complete accounts was written by Ackelrud in 2003 [67]. Some of the more articles on recent developments and applications of conjugated polymers in diodes and organic polymer lasers have been mentioned in table 1.

## 3. Overview on Organic Solid State Lighting Technology

A modern approach of conjugated polymers as peculiar light emitting materials is their suitability for achieving efficient solid state lighting (SSL) [133–143]. In contrast to conventional point source LEDs, conjugated polymer based LEDs distribute light throughout the surface area and are not restricted by their size. This brings about the possibility of having high luminance flux without glare.

### 3.1. OLED Lighting

Conjugated polymer based devices such as organic light emitting diodes (OLED) are a new light emitting medium [144,145], in which the emitting layer material of the LED is an organic compound, known as an organic light emitting diode (OLED) [146–150]. OLEDs produce light in much the same way that ordinary LEDs do, except that the positive and negative charges originate in organic compounds rather than in crystalline semiconductors. They emit light across the visible, ultraviolet and infra red wavelengths, with very high brightness and have the potential for energy efficient solutions [151–156]. From the commercial market point of view, OLEDs are promising devices for thinner, lighter, and higher-resolution displays for next generation televisions, computers, electronic books, and billboards. A lot of exciting OLED gadgets have been introduced, including the Google Nexus-One and Nexus-S, Samsung’s Jet, Wave and Galaxy-S, Nokia’s N8, E7 and C7, three WP7 phones, and several HTC phones [157].

OLEDs have the potential to outperform all other light sources. OLED is not a lamp, nor just a light source—it is a new light emitting medium without sacrificing the aesthetic appeal and essential lighting properties: lumen maintenance, sustainability, low cost and efficiency [158]. Reported record efficiencies of 110 lm/W for green light and performance targets of ongoing research and development activities focused on white emission indicate the potential of OLEDs to emerge as a solid state lighting source for a wide variety of potential applications, including ambient and technical lighting as well as signage applications, such as exit signs or logos. As an example, Osram Opto Semiconductors has introduced Orbeos, its first OLED light source, which is aimed at premium-quality functional lighting applications such as architecture, hotels and catering, offices, private homes and shops. The Orbeos OLED panel has a round lamp surface of 80 mm diameter, is 2.1 mm thick and weighs 24 g. Osram believes that these limited dimensions will ensure plenty of different usage options. The panel’s efficiency is quoted as 25 lm/W, which Osram states is better than conventional halogen lamps. It has a warm-white color temperature of 2,800 K, with a Color Rendering Index CRI of up to 80, making it suited to lighting that is “atmospheric and functional at the same time.”

According to Osram, OLEDs “rate highly with their pleasant, non-glare light” and open up totally new design possibilities for architects, lighting planners and designers, making it possible to create illuminated areas such as lit ceilings or partitions. Orbeos can be switched on and off without delay, and is continuously dimmable. Unlike LEDs, heat management is simple. The panel contains no mercury and emits no UV or infrared radiation. Its brightness level is usually 1,000 cd/m^2^ with power input of less than a watt. In ideal operating conditions it has a lifespan of around 5,000 hours.

### 3.2. OLEDs’ Lighting Benefits

Energy policies encourage technologies that can offer maximum energy savings; OLED technology falls into this category. OLEDs offer many advantages over both LCDs and LEDs. Adoption of OLED lighting has the following advantages:

- OLEDs have a significantly lower price than LCDs or plasma displays due to the fact that they can be printed onto any suitable substrate using an inkjet printer or even screen printing technologies.
- The ability of OLEDs to be printed onto flexible substrates has opened the gate to several new applications, like roll-up displays and displays embedded in fabrics.
- OLED pixels directly emit light, thus provides a greater range of colors, brightness, and viewing angle than LCDs.
- One remarkable advantage of OLEDs is the ability of color tuning.
- Energy saving potential.
- Mercury-free.
- New freedom in design.
- OLED substrates can be plastic rather than the glass used for LEDs and LCDs.
- High luminous efficacy.

### 3.3. Laser Lighting

Great advances in lighting technology have occurred during the past couple of decades [159–162]. Given the advantages of lasers over lamps in key aspects such as reliable brightness and commercial reality, many attempts have been made to develop and launch lasers in a variety of illumination and specialty lighting applications [163,164]. Conjugated polymers have been identified as a promising class of materials for laser applications owing to their high-emission efficiency, large cross sections for stimulated emission and wide spectral coverage [165–166]. Since the conjugated polymers have been prescribed as beneficial light emitting materials, it is a very obvious step to try to introduce their inherent advantages into the laser field. For laser devices, conjugated polymers work as a novel class of solid-state laser active media with great potential for lasing dynamics and optical amplification due to their broad spectra and high optical gains. In recent years, conjugated polymers have become an attractive new gain medium for lasers that are tuneable across the visible spectrum. The high gain available from conjugated polymers in the visible spectrum indicates that conjugated polymer laser are compatible with the low-cost polymer optical fibers (POF), leading to their potential use as light sources for short-haul communication networks. The broad spectra of these materials make them potential candidates for ultrafast photonics as the generation of a short laser pulse calls for a gain medium which operates over a wide range of optical energies. A major advantage of conjugated polymers is their solubility in a wide range of solvents. By dissolving the material in an appropriate solvent, the solution can be easily processed, for example by spin-coating, inkjet printing or micromolding, making mass fabrication a real prospect. First of all, it is desirable for the laser material to have the electronic structure of a four-level system so that the stimulated emission spectrum does not overlap with the ground state absorption spectrum.

Fortunately, most conjugated polymers naturally form a four-level system because structural and vibronic relaxation in the excited state shifts the energy levels. The key photophysical properties that make these polymers good as laser materials are the shift between the absorption and emission spectra that leads to low self-absorption, the high PL efficiency combined with high chromophore density, the high stimulated emission cross section and the absence of excited-state absorption in the luminescent spectral region [167,168].

Although the development of polymer lasers is at a much earlier stage than polymer LEDs, enormous progress has been made in understanding and improving the optical design, reducing threshold, and exploiting polymer properties to enable simple patterning. In 1992, Moses demonstrated the first conjugated polymer lasing using a liquid-dye laser configuration [169]. In that report the polymer MEH-PPV was used in solution and replaced commonly used dyes. Recently these polymers have become known as an attractive new gain medium for lasers and the nature of the photoexcitations in conjugated polymers and their fundamental dynamics are of great importance for optimizing lasing properties [170–172]. There are several reasons why semiconducting polymers could be attractive laser materials.

- Reducing threshold.
- Simple fabrication of microstructure.
- Semiconducting polymers and ultrafast photonics.
- Toward electrical pumping of polymer lasers.
- Low cost.

## 4. Summary and Future Prospects

The conjugated polymers’ contribution in the area of plastic electronics is leading to a variety of products with high energy efficiency and reduced environmental impact. Conjugated polymer-based materials are bringing about a revolution and paradigm shift in the optoelectronics sector, with far-reaching consequences for applications in display devices, lighting, sensing and solar energy harvesting.

Already a variety of advanced optical and electronic products based on conjugated polymers are in the market place such as light-emitting diodes, thin film transistors, photovoltaic cells, sensors, plastic lasers, and nonlinear optical systems. Yet the future of the conjugated polymer based devices holds even greater promise for an entirely new generation of ultra low cost, light-weight and flexible electronic devices, moreover, these are expected to supersede many of the existing inorganic semiconductor based devices. Have you ever imagined having a display monitor built into your clothing that can be rolled up? Have you conceived having a high-definition TV, 80 inches wide but less than a quarter-inch thick, and that consumes less power than most TVs?

These devices are not just an artist’s speculation; they might be possible in the near future with the help of OLEDs. One of the most interesting aspects of OLED is that it can be used to build transparent and flexible screens. One of its applications can be “transparent window.” By day, it would work like a common transparent plastic window. As light fades, flick a switch and it becomes a light fixture. It could cut energy costs by switching dynamically when sunlight will suffice.

## Figures and Tables

**Table 1.** Recent literature on light emitting conjugated polymers based devices.

**Year**	**First author**	**Paper title**	**References no.**
2011	Tarver, J.	Organic electronic devices with water-dispersible conducting polymers	Comprehensive Nanoscience and Technology, Chapter 4.14, 413–446. [68]
2011	Antonio, F.	π-Conjugated polymers for organic electronics and photovoltaic cell applications	Chem. Mater. 23, 733–758. [69]
2010	Schumacher, S.	Dynamics of photo excitation and stimulated optical emission in conjugated polymers: A multi scale quantum-chemistry and Maxwell-Bloch-equations approach	Phys. Rev. B 81, 245407–11. [70]
2010	Ebinazar, B.N.	Organic light emitting complementary inverters	Appl. Phys. Lett. 96, 043304–3. [71]
2010	Carlos, S.	Organic semiconductors: A little energy goes a long way	Nature Mater. 9, 884–885. [72]
2010	Cuihong, L.	Three-dimensional conjugated macromolecules as light-emitting materials	Polymer 51, 4273–4294. [73]
2010	Adam, J.M.	Power from plastic	Curr. Opin. Solid State Mater. Sci. 14, 123–130. [74]
2010	Shufen, C.	Recent developments in top-emitting organic light-emitting diodes	Adv. Mater. 22, 5227–5239. [75]
2010	Taeshik, E.	Solution-processed highly efficient blue phosphorescent polymer light-emitting diodes enabled by a new electron transport material	Adv. Mater. 22, 4744–4748. [76]
2010	Tao, R.	Blue phosphorescence materials for organic light-emitting diodes	Prog. Chem. 22, 2215–2227. [77]
2010	Jenny, C.	Organic photonics for communications	Nature. Photon. 4, 438–446. [78]
2010	Neil, W.	Conjugated polymers: Phases go their separate ways	Nature. Chem. June, 748–748. [79]
2010	Shahul, H.	Polymer light emitting diodes —A review on Materials and techniques	Rev. Adv. Mater. Sci. 26, 30–42. [80]
2009	Stefano, T.	Lighting technology: Time to change the bulb	Nature 459, 312–314. [81]
2009	Namdas, E.B.	Low threshold in polymer lasers on conductive substrates by distributed feedback nanoimprinting: Progress toward electrically pumped plastic lasers	Adv. Mater. 21, 799–802. [82]
2009	Hui, J.	Conjugated polyelectrolytes: Synthesis, photophysics, and applications	Angew. Chem. Int. Ed. 48, 4300–4316. [83]
2009	Rachel, A.S.	Block copolymers for organic optoelectronics	Macromolecules 42, 9205–9216. [84]
2008	Daniele, B.	High-performance organic field-effect transistors	Adv. Mater. 21, 1473–1486. [85]
2008	Qi, D.L.	Polymer electronic memories: Materials, devices and mechanisms	Prog. Polym. Sci. 33, 917–978. [86]
2008	Kalinowski, J.	Optical materials for organic light-emitting devices	Opt. Mater. 30, 792–799. [87]
2008	Johannes, K.F.	Poly(arylene vinylene)s	High Perform. Polym. 1, 89–137. [88]
2008	Inamul, H.R.	Recent progress in the development of polymers for white light-emitting polymer devices	Monatsh. Chem. 139, 725–737. [89]
2008	Abouelaoualim, D.	Numerical study of electrical characteristics of conjugated polymer light-emitting diodes	Semiconduct. Phys. Quantum Electron. Optoelectr. 11, 151–153. [90]
2008	Yang, X.	Saturation, relaxation, and dissociation of excited triplet excitons in conjugated polymers	Adv. Mater. 20, 1–4. [91]
2008	Murano, S.	Highly Efficient White PIN OLEDs for Lighting Applications	LED J. 40–41. [92]
2008	Sony, a.b.	a. b. Sony XEL-1:The world’s first OLED TV	www.OLED-Info.com. [93]
2007	Samuel, I.D.W.	Organic semiconductor lasers	Chem. Rev. 107, 1272–1295. [94]
2006	Friend, R.	Polymers show they’re metal	Nature 441, 37, 1–1. [95]
2006	Amarasingh, D.	Broadband solid state optical amplifier based on a semiconducting polymer	Appl. Phys. Lett. 89, 2011–2019. [96]
2006	Roger, J.M.	Electrochromic organic and polymeric materials for display applications	Displays 27, 2–18. [97]
2005	Danilo, D.	Electrochemiluminescence from organic emitters	Chem. Mater. 17, 1933–1945. [98]
2005	Service, R.F.	Organic LEDs look forward to a bright, white future	Science 310, 1762–1763. [99]
2005	David, G.L.	Laser-assisted patterning of conjugated polymer light emitting diodes	Org. Electr. 6, 221–228. [100]
2005	Stuart, S.	Case study: Cambridge Display Technology Ltd.	University of Cambridge Centre for Technology Management, pp. 1–19. [101]
2004	Andrade, B.W.D.	White organic light emitting devices for solid state lighting	Adv. Mater. 16, l585–l595. [102]
2004	Kulkarni, A.P.	Electron transport materials for organic light-emitting diodes	Chem. Mater. 16, 4556–4573. [103]
2004	Forrest, S.R.	The path to ubiquitous and low-cost organic electronic appliances on plastic	Nature 428, 911–918. [104]
2004	Josemon, J.	Progress towards stable blue light-emitting polymer	Curr. Appl. Phys. 4, 339–342. [105]
2004	Ifor, D.W.S.	Laser physics: Fantastic plastic	Nature 429, 709–711. [106]
2004	Ifor, D.W.S.	Towards polymer lasers and amplifiers ultrafast photonics	Ultrafast Phot. Taylor & Francis, 291–304. [107]
2004	Hiroyuki, S.	Organic light-emitting materials and devices for optical communication technology	J. Photochem. Photobiol. 166, 155–161. [108]
2004	John, K.B.	Developments in organic displays	Mater. Today 7, 42–46. [109]
2004	Asawapirom, U.	Materials for polymer electronics applications—Semiconducting polymer thin films and nanoparticles	Macromol. Symp. 212, 83–91. [110]
2002	Hong, K.S.	Light-emitting characteristics of conjugated polymers	Adv. Polym. Sci. 158, 193–243. [111]
2002	David, B.	Semiconducting polymer LEDs	Mater. Today 5, 3032–3039. [112]
2002	Hung, L.S.	Recent progress of molecular organic electroluminescent materials and devices	Mater. Sci. Eng. R 39, 143–222. [113]
2002	Köhler, A.	Fluorescence and phosphorescence in organic materials	Adv. Eng. Mater. 4, 453–459. [114]
2002	Brabec, C.J.	A low-bandgap semiconducting polymer for photovoltaic devices and infrared emitting devices	Adv. Funct. Mater. 12, 709–712. [115]
2002	Vander, H.J.W.	Electronic and optical excitations in crystalline conjugated polymers	Phys. Rev. B 66, 035206:1–035206:7. [116]
2001	Heeger, A.J.	Nobel Lecture—Semiconducting and metallic polymers—The fourth generation of polymeric materials	Rev. Modern Phys. 73, 681–700. [117]
2001	McDiarmid, A.G.	Nobel lecture—“Synthetic metals”—a novel role for organic polymers	Rev. Modern Phys. 73, 701–712. [118]
2001	Shirakawa, H.	Nobel lecture: The discovery of polyacetylene film—The dawning of an era of conducting polymers	Rev. Modern Phys. 73, 713–718. [119]
2001	Philip, B.	A happier marriage	Nature, Nature News, 010201–3. [120]
2001	Scherf, U.	Conjugated polymers: Lasing and stimulated emission	Curr. Opin. Solid State Mater. Sci. 5, 143–154. [121]
2001	Friend, R.H.	Conjugated polymers. New materials for optoelectronic devices	Pure Appl. Chem. 73, 425–430. [122]
2001	Lee, C.H.	Photoluminescence and electroluminescence of vacuum-deposited poly(p-phenylene) thin film	Synth. Met. 117, 75–79. [123]
2001	Liming, D.	Effect of forster energy transfer and hole transport layer on performance of polymer light-emitting diodes	Macromolecules 34, 9183–9188. [124]
2000	Philip, B.	Let there be more light	Nature, Nature News, 000217–11. [125]
2000	Kranzelbinder, G.	Organic solid-state lasers	Rep. Prog. Phys. 63, 729–762. [126]
2000	Ullrich, M.	The electroluminescence of organic materials	J. Mater. Chem. 10, 1471–1507. [127]
2000	Tien, Y.L.	Electroluminescent polymeric materials	Curr. Sci. 78, 1352–1357. [128]
2000	Marai, F.	Photoluminescence and electroluminescence investigations in PEPPV and its derivatives	Synth. Met. 114, 255–259. [129]
2000	Markus, G.	Improving the performance of doped π-conjugated polymers for use in organic light-emitting diodes	Nature 405, 661–665. [130]
2000	Sun, R.	High PL quantum efficiency of poly(phenylene vinylene) systems through exciton confinement	Synth. Met. 111–112, 595–602. [131]
2000	Bernius, M.T.	Progress with light-emitting polymers	Adv. Mater. 12, 1737–1750. [132]
